# The immune suppressive properties of damage associated molecular patterns in the setting of sterile traumatic injury

**DOI:** 10.3389/fimmu.2023.1239683

**Published:** 2023-08-15

**Authors:** Emily Horner, Janet M. Lord, Jon Hazeldine

**Affiliations:** ^1^ Institute of Inflammation and Ageing, University of Birmingham, Birmingham, United Kingdom; ^2^ National Institute for Health Research Surgical Reconstruction and Microbiology Research Centre, Queen Elizabeth Hospital Birmingham, Birmingham, United Kingdom

**Keywords:** critical care, damage associated molecular patterns, immunosuppression, innate immunity, trauma

## Abstract

Associated with the development of hospital-acquired infections, major traumatic injury results in an immediate and persistent state of systemic immunosuppression, yet the underlying mechanisms are poorly understood. Detected in the circulation in the minutes, days and weeks following injury, damage associated molecular patterns (DAMPs) are a heterogeneous collection of proteins, lipids and DNA renowned for initiating the systemic inflammatory response syndrome. Suggesting additional immunomodulatory roles in the post-trauma immune response, data are emerging implicating DAMPs as potential mediators of post-trauma immune suppression. Discussing the results of *in vitro, in vivo* and *ex vivo* studies, the purpose of this review is to summarise the emerging immune tolerising properties of cytosolic, nuclear and mitochondrial-derived DAMPs. Direct inhibition of neutrophil antimicrobial activities, the induction of endotoxin tolerance in monocytes and macrophages, and the recruitment, activation and expansion of myeloid derived suppressor cells and regulatory T cells are examples of some of the immune suppressive properties assigned to DAMPs so far. Crucially, with studies identifying the molecular mechanisms by which DAMPs promote immune suppression, therapeutic strategies that prevent and/or reverse DAMP-induced immunosuppression have been proposed. Approaches currently under consideration include the use of synthetic polymers, or the delivery of plasma proteins, to scavenge circulating DAMPs, or to treat critically-injured patients with antagonists of DAMP receptors. However, as DAMPs share signalling pathways with pathogen associated molecular patterns, and pro-inflammatory responses are essential for tissue regeneration, these approaches need to be carefully considered in order to ensure that modulating DAMP levels and/or their interaction with immune cells does not negatively impact upon anti-microbial defence and the physiological responses of tissue repair and wound healing.

## 1 Introduction

Accounting for an estimated 16% of the global burden of disease (1), and the leading cause of death in individuals aged ≤44 years (2), traumatic injuries are a significant cause of morbidity and mortality. However, due in part to advances in trauma medicine, most notably in the management of coagulopathy where haemorrhage-induced deaths have fallen by more than 12% since the 1980s (3), the likelihood of patients surviving the initial effects of a severe trauma is increasing (4). Consequently, it is the onset of secondary complications such as multiple organ failure (MOF), multiple organ dysfunction syndrome (MODS) and nosocomial infections that are emerging as major determinants of clinical outcomes. Indeed, when compared to those with non-eventful episodes, trauma patients who develop MOF, MODS or hospital-acquired infections (HAIs) experience increased lengths of intensive care unit stay, require prolonged mechanical ventilation, report poorer physical and psychological recovery, and are less likely to return to work (5–10). Of these secondary complications, HAIs are highly prevalent (5, 6, 11–14) and remain a significant clinical challenge, with one recent study estimating that the prevention of pneumonia amongst hospitalised trauma patients would reduce the incidence of end organ dysfunction, mortality and the need for mechanical ventilation by 22.1%, 7.8% and 6.8% respectively (13).

Two opposing and concurrent syndromes underpin the immune and inflammatory response to major trauma. With the intention of localising and eliminating endogenous stressors, and promoting wound repair, a systemic inflammatory response syndrome (SIRS) is triggered within minutes of injury (15). Associated with such physiological changes as tachycardia and tachypnea, the SIRS response is characterised by elevated levels of circulating pro-inflammatory cytokines and immune cell activation. To counteract this state of systemic hyperinflammation, the SIRS response is accompanied by a compensatory anti-inflammatory response syndrome (CARS) (16). Defined in part by raised circulating levels of anti-inflammatory cytokines, lymphocyte apoptosis and decreased cytokine production by endotoxin challenged monocytes, the concomitant CARS response aims to restore immunological homeostasis (17). Over the past decade, it has become increasingly evident that the kinetics of the simultaneous SIRS and CARS responses markedly influence the clinical trajectories of hospitalised trauma patients, with robust, dysregulated and prolonged SIRS/CARS responses associated with delayed physical recovery, organ dysfunction, an increased risk of nosocomial infections and mortality (16, 18, 19). Furthermore, failure to resolve trauma-induced inflammatory responses can lead to the development of persistent inflammation, immunosuppression and catabolism syndrome (PICS). Detected in >40% of patients with chronic critical illness, PICS has been suggested to have replaced MOF as the predominant phenotype of chronic critical illness (20–23). Characterised by lymphopenia, neutrophilia, a persistent acute phase response and ongoing protein catabolism, the development of PICS predisposes to recurrent HAIs, poor wound healing and a greater need for post-discharge rehabilitation in long-term care facilities (20–24).

Hypothesising that factors released into the circulation or present in wound fluids may modulate the local and systemic immune response to sterile injury, several groups have investigated the phenotypic and functional consequences of culturing immune cells isolated from healthy subjects or mice with plasma, serum or wound fluids obtained from their injured counterparts (25–28). Compared to control samples, plasma acquired from trauma patients at day 1 post-injury, wound fluids obtained either 3- or 6-hours post-surgery or from closed drains in injury sites, and serum collected from mice within 30 minutes of injury, were found to promote the expansion of both CD8^+^ T cells and CD11c^+^ Natural Killer (NK) cells, and to suppress the anti-microbial responses of neutrophils and monocytes (25–28). These are all features reminiscent of the trauma associated SIRS and CARS responses (15, 29–31). Whilst recognising that a multitude of factors are present in the circulation of trauma patients, the authors suggested a potential role for damage associated molecular patterns (DAMPs) in mediating the effects they observed (25, 26, 28). Detected in the circulation of major trauma patients in the minutes, hours and days following injury (30–33), DAMPs, also known as alarmins, are a heterogeneous collection of cytosolic, nuclear and mitochondrial-derived lipids, proteins and DNA. Passively released from damaged/necrotic tissue, and actively secreted by immune cells (34, 35), DAMPs are detected primarily by pathogen recognition receptors (PRRs), particularly members of the Toll-like receptor (TLR) family (36) **(** **Table 1** **)**. To date, much attention has focussed on the immune stimulatory properties of DAMPs (69, 70), with studies showing that *in vitro* exposure to mitochondrial, nuclear and cytosolic-derived DAMPs can trigger ROS generation, degranulation, formation of extracellular traps and the production of pro-inflammatory cytokines by neutrophils, monocytes, macrophages and invariant NKT cells (33, 71–75). Similar pro-inflammatory responses have also been observed in animal studies, where administration of mitochondrial-derived DAMPs (mtDAMPs) has been shown to increase plasma cytokine concentrations and activate neutrophils, systemic responses that result in immune-mediated organ injury (33, 76). Importantly, these observations are aligned with the results of prospective studies of trauma patients, where elevated circulating concentrations of mitochondrial-derived DNA (mtDNA) and the nuclear-derived DAMP high mobility group box-1 (HMGB-1) are associated with the development of SIRS and MODS (76–80).

**Table 1:** Summary of the immune suppressive properties of DAMPs.

DAMP	Receptor	Immune suppressive properties	References
Cytosolic			
*Heme*	TLR4	Decreases TLR2 and TLR4 expression on neutrophils. Inhibits neutrophil ROS production. Suppresses macrophage phagocytic activity.	(27, 37)
*HSPs*	TLR4, TLR9	Reduces LPS-induced TNF production by monocytes. Inhibits NF-κB signalling. Impairs DC maturation. Enhances immune suppressive activity of Tregs.	(28, 38–40)
*S100 proteins*	TLR2, TLR4, RAGE	Induces endotoxin tolerance in monocytes. Inhibits p38 MAPK signalling in monocytes. Decreases antigen presentation by DCs. Suppresses neutrophil ROS production. Chemoattractant for MDSCs.	(41–46)
Nuclear			
*CIRP*	TLR4, TREM-1	Induces endotoxin tolerance in macrophages. Promotes M2 polarisation in macrophages.	(47)
*IL-33*	ST2	Stimulates IL-5 production and arginase-1 expression in neutrophils. Promotes expansion of ILC2 cells. Induces immune suppressive functions in neutrophils. Drives expansion of IL-10 secreting M2 macrophages. Promotes accumulation of Tregs.	(48–50)
*HMGB-1*	TLR2, TLR4, TREM-1, RAGE	Suppresses NF-B signalling. Reduces pro-inflammatory cytokine production by macrophages. Decreases macrophage phagocytic activity. Inhibits neutrophil ROS production. Promotes survival, migration and suppressive activity of Tregs. Activates and prolongs survival of MDSCs.	(51–56)
Mitochondrial			
*ATP/adenosine*	A_2A_	Inhibits neutrophil phagocytosis, ROS production, degranulation and cytokine production. Suppresses T cell migration and IFN-γ production. Decreases T cell priming by DCs. Inhibits NK cell cytotoxicity. Induces endotoxin tolerance in macrophages.	(57–60)
*mtDNA*	TLR9	Reduces pro-inflammatory cytokine production by LPS-challenged monocytes. Induces IRAK-M expression in monocytes. Inhibits neutrophil chemotaxis, phagocytosis and bacterial killing. Suppresses activation of CTTN. Promotes IL-10 production by DCs.	(35, 61, 62, 63, 64)
*mtFPs*	FPR1, FPR2	Decreases neutrophil calcium fluxes. Suppresses neutrophil chemotaxis and ROS production. Downregulates GPCR expression. Inhibits NET formation.	(30, 65–68)

ATP, Adenosine triphosphate; CIRP, Cold-inducible RNA binding protein; CTTN, Cortactin; DAMPs, Damage associated molecular patterns; DC, Dendritic cell; FPR, Formyl peptide receptor; GPCR, G-protein coupled receptor; HMGB-1, High mobility group box-1; HSP, Heat shock protein; IFN, Interferon; IL, Interleukin; ILC2, Group 2 innate lymphoid cells; IRAK-M, Interleukin 1 receptor associated kinase-M; LPS, Lipopolysaccharide; MAPK, Mitogen activated protein kinase; MDSC, Myeloid derived suppressor cell; mtDNA, Mitochondrial-derived DNA; mtFPs, Mitochondrial-derived formyl peptides; NF-κB, Nuclear factor-κβ; RAGE, Receptor for advanced glycation endproducts; ROS, Reactive oxygen species; ST2, Suppression of tumorigenicity; TLR, Toll-like receptor; Treg, Regulatory T cell; TREM-1, Triggering receptor expressed on myeloid cells-1.

Interestingly, in addition to their widely accepted role as instigators of the SIRS response, data are emerging that implicates DAMPs as potential mediators of the CARS response, with *in vitro* studies demonstrating that neutrophils and monocytes pre-treated with cytosolic and/or mitochondrial-derived DAMPs exhibit a state of functional tolerance upon secondary stimulation with such inflammatory agonists as bacterial endotoxins and chemokines (30, 35, 41, 61, 65, 81–83) **(** **Table 1** **)**. This DAMP-induced immune suppression may also extend to the adaptive arm of the immune system, where it has been suggested that, by promoting interleukin-4 production and T helper 2 immunity, DAMPs may suppress pro-inflammatory T helper 1 responses (81, 84). Alongside DAMPs that are passively released from damaged/necrotic tissue, a novel class of *de novo* synthesised DAMPs with immunosuppressive properties, termed suppressing/inhibiting inducible DAMPs (SAMPs), have recently been described. Produced by activated leukocytes, SAMPs, which include prostaglandin E2 and Annexin A1, have been suggested to promote exaggerated anti-inflammatory and hyper-resolution responses that culminate in systemic immunosuppression (82). Thus, exaggerated and/or persistent DAMP driven resolution responses may predispose the hospitalised trauma patient to the development of secondary infections (82). Supporting this idea, a study of 166 adult trauma patients found plasma levels of both mtDNA and cell-free nuclear DNA (used as a generic readout for cell damage and death) were significantly higher at hospital admission in subjects who subsequently developed an infection within 28 days (31). Moreover, the authors reported that plasma concentrations of the cytosolic DAMP, heat shock protein-70 (HSP-70), and nuclear DNA (nDNA) both correlated negatively with the expression of HLA-DR on monocytes (31), a marker of immune competence whose downregulation is associated with a higher risk of nosocomial infections in critically ill patients (85–87).

Proposed as a potential explanation for the failures of randomised clinical trials that have tested the practicalities and benefits of immunomodulatory interventions for the treatment of critically-ill patients, compartmentalisation is a concept that refers to how immune responses differ between anatomical sites within the body (88). Examples of compartmentalisation include the varied phenotypic and morphological features described for neutrophils isolated from the blood and lungs of patients with acute respiratory distress syndrome (ARDS) (89), and the different mediators that drive the phagocytic dysfunction of peripheral blood and alveolar residing neutrophils in critically ill patients (90). To date, almost all studies that have investigated DAMP-induced immunosuppression in an *ex vivo* setting have examined the functional and phenotypic features of circulating leukocytes. Thus, whilst one may assume that DAMPs, whose concentrations at sites of tissue damage exceed those measured in the circulation (91), would also suppress localised immune responses, there is a current paucity of information on whether DAMPs can modulate the behaviours of tissue resident immune cells. That said, with results from a small number of studies demonstrating a role for DAMPs in the impaired *ex vivo* responses of macrophages and T cells (47, 48, 92), and in promoting the expansion of tissue resident myeloid-derived suppressor cells (MDSCs) (93), it does appear that the release of DAMPs from damaged tissue may contribute to the immune dysfunction that develops in different tissue spaces following major trauma.

Bringing together data generated from *in vitro, in vivo* and *ex vivo* studies, the purpose of this review is to summarise our current understanding of DAMP-induced immunosuppression in the setting of sterile injury. The immune suppressive properties of cytosolic, nuclear and mitochondrial-derived DAMPs will be discussed, and where identified, their underlying mechanisms described. Furthermore, with the aim of reducing the susceptibility of critically-ill patients to HAIs, we will highlight the therapeutic strategies that are under consideration as potential approaches for preventing or reversing DAMP-induced immunosuppression.

## 2 Immunosuppressive properties of DAMPs

### 2.1 Cytosolic-derived DAMPs

#### 2.1.1 Heme

Heme is an iron containing porphyrin synthesised in the cytosol and mitochondria of erythrocytes. Acting as the prosthetic group for such hemoproteins as haemoglobin, myoglobin and cytochrome P450, heme plays an essential role in a range of biological processes, which include oxygen transport and storage, electron transfer and cellular metabolism (94). However, when released from hemoproteins, unbound heme promotes oxidative stress, inflammation and tissue injury via its ability to catalyse the formation of highly reactive oxygen free radicals, amplify TLR-mediated immune responses and activate the complement cascade (94–98). Thus, in the setting of critical illness, links have been suggested between elevated concentrations of cell-free heme, the SIRS response and organ injury (99). To counteract its cytotoxicity, circulating levels of cell-free heme are regulated primarily by the endogenous heme-binding plasma protein hemopexin (100), with this scavenging system supported by albumin, α-2-macroglobulin and haptoglobin, three heme/haemoglobin binding plasma proteins that assist in the neutralisation, recycling and removal of free heme and iron (94, 95, 99).

Compared to healthy controls, significantly higher concentrations of free heme have been detected in plasma samples obtained from trauma patients at day 1 post-injury (27). Interestingly, insights from a murine model of non-lethal blunt liver trauma suggest a rapid emergence and clearance of circulating heme post-injury, with serial measurements across a 24-hour window (0.5, 1, 4 and 24 hours) detecting a significant trauma-induced increase in plasma heme concentrations only in samples obtained within 30 minutes of injury (27). This apparent discordance in the kinetics of heme release, and its persistence, between humans and mice could potentially reflect species differences in the scavenging of free heme. For instance, in murine models of trauma and burn wound infections, significant increases in plasma levels of hemopexin and haptoglobin have been reported (27, 101, 102), whereas in cohorts of trauma and burns patients, plasma concentrations of hemopexin, haptoglobin and α-2-macroglobulin were found to be comparable to or lower than the levels detected in healthy controls (27, 101–103). In line with these observations, a study of fifty stably resuscitated trauma haemorrhage patients found that within 2-3 hours of resuscitation, plasma hemopexin levels were significantly lower than those of free heme, resulting in a hemopexin:free heme ratio of <1 (37). In addition to the act of trauma itself, medical interventions may influence circulating heme levels, with studies reporting long-term storage of leukoreduced red blood cells resulted in heme release (37, 104, 105). Consequently, when compared to fresh red blood cells, resuscitation with stored red blood cells results in significantly elevated circulating concentrations of free heme (37).

Results of *in vitro* and *in vivo* studies have provided direct evidence of the immune suppressive properties of heme (27, 37). In a murine model of *Staphylococcus aureus* (*S.aureus*) challenge, Lee et al. recovered significantly higher bacterial loads from the bronchoalveolar lavage fluid samples of mice that had been administered heme prior to infection (27), whilst impaired bacterial clearance was reported in hemopexin knockout mice challenged with *S.aureus* following a liver crush injury (27). Moreover, scavenging free heme via systemic administration of hemopexin in mice, either following blunt liver trauma, or prior to resuscitation with stored red blood cells, was shown to improve the clearance rates of *S.aureus* and *Pseudomonas aeruginosa* (27, 37). At the cellular level, data points towards suppression of the innate immune response as being the mechanism by which heme treatment delays the elimination of invading pathogens, with analysis of circulating neutrophils isolated from heme treated mice revealing significantly reduced surface expression of TLR2 and TLR4 (27). Moreover, culturing neutrophils from naïve mice in the presence of serum collected from mice within 30 minutes of a liver crush injury, when circulating heme levels peak, reduced ROS production in response to *S.aureus* challenge (27). As well as neutrophils, heme treatment has been reported to inhibit the phagocytic activity of macrophages by 90% (37). Interestingly, this inhibitory effect appears to be mediated by a heme-induced release of HMGB-1 by activated macrophages since treating macrophages with an anti-HMGB-1 blocking antibody prior to heme exposure restored their phagocytic activity to a level comparable to that of untreated controls (37).

Due to its well-established cytotoxic, pro-inflammatory and immune amplifying properties, cell-free heme has previously been discussed as a potential therapeutic target for alleviating systemic inflammation and organ injury in critically-ill patients. Heme scavenging via hemopexin therapy and the prevention of heme-induced activation of TLR4 are two examples of strategies considered to date (101, 105). Now, with data implicating heme as a potential mediator of trauma-induced immunosuppression, the idea that modulating the circulating levels of cell-free heme could reduce patient susceptibility to HAIs has been raised (27). However, our understanding of cell-free heme in the setting of traumatic injury, and its relationship with infections, must improve to test the feasibility of such a strategy. As mentioned above, animal-based studies suggest that heme levels peak within 30 minutes of injury, with levels returning to those found in uninjured controls within 4 hours (27). Currently, our knowledge of trauma-induced heme release in humans is limited to a single study whose sample acquisition began post-hospital admission, suggesting that the levels of heme reported to date may not reflect peak concentrations (27). Moreover, no study to our knowledge has examined whether a relationship exists between circulating levels of heme, hemopexin and/or haptoglobin, and the development of infections in hospitalised trauma patients. Finally, given the differences that appear to exist between rodents and humans in how trauma impacts the levels of cell-free heme and/or its scavenger proteins (27, 101–103), using murine models to test potential therapeutic strategies for counteracting heme-induced immunosuppression in human patients may be inappropriate.

#### 2.1.2 Heat shock proteins

A heterogeneous family of proteins, HSPs are molecular chaperones implicated in protein folding, stabilisation and transport (106). Constitutively expressed, and induced by such stressors as heat shock and UV radiation, HSPs are released into the extracellular environment passively, from necrotic tissue, or actively via the non-classic protein release pathway, where they are secreted as free proteins or packaged within extracellular vesicles (107, 108). Linked to the pathology of several chronic inflammatory conditions, HSPs are highly immunogenic, with studies demonstrating their capacity to stimulate cytokine production by monocytes and macrophages (107, 109), promote the maturation of dendritic cells (107) and activate the complement system (110).

Positively associated with injury severity scores (ISS), and indices of illness severity (e.g. sequential organ failure assessment score and simplified acute physiology score 3), significantly higher concentrations of HSP60, HSP70, HSP72 and/or HSP90 alpha have been measured in the circulation of polytrauma and traumatically brain-injured (TBI) patients when compared to healthy controls (31, 111–114). Correlating positively with systemic concentrations of interleukin (IL)-6 and IL-8, elevated levels of HSPs have been detected within minutes of injury and persist for up to ten days (31, 112). Intracellular expression of HSPs are also increased post-trauma, with an injury-induced induction of HSP27, HSP32, HSP60, HSP70 and HSP90 reported in neutrophils and/or monocytes isolated from major burns and trauma patients (115–117). In accordance with their pro-inflammatory nature, prospective studies have measured significantly higher concentrations of HSP60 at hospital admission (118) and HSP70 at 24- and 48-hours post-injury (111) in trauma patients that subsequently develop acute lung injury or MODS respectively. Interestingly, and suggesting a dual inflammatory role for HSPs, Ren et al. detected significantly higher serum concentrations of HSP70 in infected trauma patients 60-90 hours post-injury when compared to their non-infected counterparts (113), whilst a study of 166 adult trauma patients reported plasma HSP70 levels at hospital admission were negatively associated with leukocyte HLA-DR expression (31). Thus, a role for HSPs in mediating the post-injury CARS response has been proposed (28, 119), a theory supported by a body of literature that demonstrates the immune suppressive properties of HSPs.

Using TLR-induced pro-inflammatory cytokine production as a readout of anti-microbial capacity, HSPs have been shown to induce functional tolerance in innate phagocytic cells. Compared to untreated controls, primary human monocytes, or differentiated THP-1 cells, treated with HSP70 exhibit impaired tumour necrosis factor alpha (TNF) production in response to stimulation with lipopolysaccharide (LPS), *S.aureus* peptidoglycan or *Salmonella typhimurium* flagellin (28, 38, 83, 120). Mechanistically, the HSP70-induced impairment in TNF production triggered by LPS stimulation has been attributed to reduced gene transcription, with studies demonstrating HSP70 treatment inhibits activation of the nuclear factor-κβ (NF-κβ) signalling pathway and promotes enrichment of the transcriptional repressors heat shock transcription factor 1 and constitutive HSE-binding factor at the TNF promoter (38, 83). These tolerance-inducing properties of HSP70 contradict data showing that this protein can stimulate monocytes/macrophages to secrete TNF (107, 109). Addressing these contrasting observations, studies have suggested that endotoxin contamination within HSP70 preparations may explain the cytokine-inducing capacity assigned to this DAMP (38, 121). In addition to innate responses, HSPs exert suppressive effects on the adaptive arm of the immune system. Culminating in reduced T cell responses (122), HSP70 treatments have been shown to (1): enhance the immunosuppressive activity of regulatory T cells (39) (2); induce a tolerogenic phenotype in monocyte-derived dendritic cells (DC) (122) (3); impair DC maturation (40) (4); suppress the formation of T helper 1 cells (123). Thus, given the abundance of data demonstrating immune suppressive properties for HSPs, and the suggestion that LPS contamination of preparations may explain their reported pro-inflammatory nature, the idea that HSPs are “DAMPERs” rather than DAMPs appears more likely (124).

#### 2.1.3 S100 proteins

Located within the cytoplasm of leukocytes, microglia and astrocytes, S100 proteins are a family of calcium binding proteins involved in the regulation of cellular proliferation, metabolism and division (125, 126). Detected by the PRRs TLR2 and TLR4, and the receptor for advanced glycation endproducts (RAGE), elevated levels of S100A8, S100A9 and S100B have been detected in the circulation of TBI, polytrauma and burns patients at hospital admission and persist for up to 7 days (41, 127–129). Positively correlated with ISS, percentage total body surface area (TBSA) burn and Glasgow coma scale score (41, 129–131), prospective studies have reported significantly higher levels of S100 proteins as early as day one of injury in patients who subsequently experience poor clinical outcomes such as mortality or long-term disability (41, 127, 130–134). By augmenting the SIRS responses, the anti-apoptotic (135) and pro-inflammatory properties of S100 proteins, which include the activation of neutrophils (136), the amplification of TNF production by monocytes (137) and the promotion of leukocyte transepithelial migration (138), offer potential explanations for how injury-induced elevations in S100 proteins may contribute to poor clinical trajectories.

Studies that have investigated the immune modulatory functions of S100 proteins have shown S100A8, S100A9 and calprotectin, an S100A8/A9 heterodimer, possess immune tolerising properties. In an elegant series of experiments, Austermann et al, who measured acutely and persistently elevated serum concentrations of calprotectin in polytrauma and burns patients, uncovered the molecular mechanisms by which S100A8/A9 inhibits monocyte function (41). Resulting in impaired LPS-induced TNF production, the authors demonstrated that prior exposure of monocytes to calprotectin led to heterochromatin formation at the TNF promoter (41). Underlying this epigenetic modification was a calprotectin-induced recruitment to the TNF promoter of G9a, a methyltransferase that suppresses gene transcription via demethylation of histone H3 on lysine K9 (41). In line with this finding, other studies have shown that pre-treating macrophages or monocytes with S100A8 alone can attenuate pro-inflammatory cytokine production to bacterial challenge, with this induction of tolerance a consequence of reduced activation of the P38 mitogen activated protein kinase (MAPK) signalling pathway following ligation of TLR2/TLR4 (42). In addition to inhibiting monocyte function, S100A8, S100A9 and/or calprotectin have been reported to (1): suppress T cell priming by reducing antigen presentation by DCs (43) (2); inhibit neutrophil chemotaxis (44, 139) (3); suppress LPS and phorbol 12 myristate 13 acetate (PMA)-induced ROS production by neutrophils, with this inhibition attributed to an S100A8 and S100A9 mediated production of adenosine metabolites (140). As well as direct effects on immune cells, raised concentrations of S100 proteins at sites of tissue damage may create a localised immune suppressive environment as both S100A8 homodimers and calprotectin have been shown to act as chemoattractants for MDSCs (45, 46). Through binding to carboxylated N-glycans on such cell surface receptors as RAGE, calprotectin triggers NF-κβ signalling in MDSCs, with MDSC activation resulting in the synthesis and secretion of S100A8/A9 heterodimers (45). Thus, a post-injury vicious cycle of immunosuppression could be established, in which S100 proteins from damaged tissue not only directly suppress the function of infiltrating leukocytes, but recruit MDSCs, whose subsequent activation and production of calprotectin exacerbates existing immune dysfunction and promotes additional immunosuppression.

Interestingly, in the only study to our knowledge to have investigated the relationship between circulating calprotectin and infectious episodes in hospitalised trauma patients, Joly and colleagues found that when compared to their non-infected counterparts, patients who developed a HAI exhibited a smaller increase in plasma calprotectin concentrations during the first five days of hospitalisation (141). If calprotectin exerts suppressive effects on innate and adaptive immune cells, *how can a smaller increase in its circulating levels make patients prone to infection?* Since calprotectin is secreted by activated neutrophils and monocytes (142, 143), the authors speculated that their findings may reflect a sign of systemic immune exhaustion and thus decreased anti-microbial activity, thereby increasing patient susceptibility to HAIs (141).

### 2.2 Nuclear-derived DAMPs

#### 2.2.1 Cold-inducible RNA binding protein

A regulator of mRNA translation and cell proliferation, CIRP is an RNA chaperone protein, which in the steady state resides in the nucleus (144, 145). In times of stress, triggered by mild hypothermia, hypoxia or oxidation, CIRP is transported into the cytosol, where it is actively secreted, either via the lysosomal pathway or through gasdermin D membrane channels (145–148), or released passively into the extracellular space as a consequence of necroptosis (149).

Signalling through TLR4, triggering receptor expressed on myeloid cells-1 (TREM-1) and NF-κβ (147, 150–153), extracellular CIRP (eCIRP) has been shown to: (1) induce TNF production, ROS generation and extracellular trap formation by macrophages and monocytes (147, 151, 153–155); (2) promote ICAM-1 expression and extracellular trap generation by neutrophils (152, 156, 157); (3) stimulate differentiation of CD4^+^ T cells to Th1 cells (150); (4) enhance the cytotoxic profile of CD8^+^ T cells (150). Unsurprisingly therefore, in *in vivo* models of sepsis or haemorrhage, eCIRP amplifies systemic inflammatory responses that culminate in organ dysfunction, injury and increased mortality (147, 148, 150, 151, 153, 158). Interestingly, eCIRP may also exacerbate inflammation by delaying the resolution of inflammatory responses. In a series of *in vitro* co-culture experiments, Chen et al. demonstrated that eCIRP-induced NETs significantly inhibited macrophage efferocytosis, with this suppression a consequence of NET associated neutrophil elastase cleaving the integrins α_v_β_3_ and α_v_β_5_ from the macrophage surface (157).

eCIRP has been detected in the circulation of critically-ill ICU patients and in the serum of rats subjected to haemorrhagic shock and cecal ligation and puncture (47, 147). As reported for other DAMPs, innate immune cells pre-exposed to CIRP exhibit impaired functional responses upon secondary stimulation. Compared to vehicle controls, pre-treatment of peritoneal macrophages isolated from healthy mice with CIRP for 24 hours resulted in significantly reduced IL-6 and TNF production upon subsequent LPS challenge (47). This induction of tolerance was confirmed *ex vivo*, with macrophages obtained from mice injected with CIRP exhibiting impaired IL-6 production when stimulated with LPS *in vitro* (47). Mechanistically, this state of endotoxin tolerance was attributed to an eCIRP-mediated activation of STAT3, which was downstream of eCIRP binding to the IL-6 receptor (IL-6R) (47). Indeed, pharmacological inhibition of STAT3 signalling or the use of anti-IL-6R antibodies reversed eCIRP-induced macrophage endotoxin tolerance (47). Pointing towards a role in modulating macrophage polarisation, eCIRP treatment of RAW264.7 cells increased their expression of arginase-1 and CD206 (47). The up-regulation of these markers suggests eCIRP promotes polarisation of macrophages towards an M2 anti-inflammatory phenotype (47). Interestingly, besides inflammatory cytokines, eCIRP treatment of RAW264.7 cells results in secretion of HMGB-1 (147), a DAMP that also possesses endotoxin tolerising properties (51). Thus, a feedback loop of DAMP-induced immunosuppression, initiated by eCIRP and reinforced by HMGB-1, could be established at sites of tissue injury that would result in localised immunosuppression and impaired bacterial clearance.

Given its pro-inflammatory properties and associations with organ dysfunction in models of systemic inflammation, eCIRP has been discussed as a potential therapeutic target for the treatment of sepsis (148, 159). Lending support to this idea, *in vitro* and *in vivo* studies have shown antagonists of CIRP signalling that include C23, which binds to the TLR4-MD2 receptor complex, and M3, which blocks the interaction of eCIRP with TREM-1, can suppress macrophage activation, reduce circulating concentrations of pro-inflammatory cytokines, attenuate organ injury and improve survival rates (151, 153, 158). With a link now emerging between eCIRP, the IL6R and immunosuppression, a call for studies to investigate whether targeting this pathway could negate eCIRP-induced immune tolerance in critically-ill patients has been made, with the suggestion that research should begin by testing small peptide antagonists that target the interaction between eCIRP and IL6R in preclinical models of immune tolerance and secondary infections (47).

#### 2.2.2 Interleukin -33

Constitutively expressed by epithelial, endothelial and stromal cells, and induced in macrophages and DCs in times of inflammation and infection, IL-33 is a member of the IL-1 cytokine superfamily (160, 161). Residing in the nucleus, IL-33 is a dual function protein, acting as a transcriptional repressor and cytokine (160). When released into the extracellular environment, either as a result of cellular damage or active secretion, IL-33 binds to its receptor suppression of tumorigenicity (ST2) (160, 161). Expressed by such cells as mast cells, eosinophils, Th2 cells, group 2 innate lymphoid cells (ILC2) and NK cells, activation of ST2 signalling pathways results in cytokine production, degranulation and T cell polarisation (162, 163).

In a prospective study of 472 blunt trauma patients admitted to ICU, Xu and colleagues detected an early (upon hospital admission) and persistent (up to day 7) post-injury elevation in circulating IL-33 levels (49), a finding that was confirmed in a subsequent independent study of 14 polytrauma patients, in whom plasma IL-33 concentrations were significantly higher 6 to 72 hours post-injury when compared to a mixed control population of monotrauma patients and healthy controls (164). Interestingly, in burns patients, a significant injury-induced decrease in serum IL-33 levels has been reported, with this reduction evident on the day of hospital admission and persisting for up to 28 days (165). Whilst it is debatable whether circulating IL-33 levels are influenced by injury severity (49, 164, 166), differences have been observed in IL-33 concentrations between patients with varied clinical outcomes. For example, in the abovementioned study of 14 polytrauma patients, plasma IL-33 levels at hospital admission were reported to be positively associated with SIRS and Denver MOF scores at day 3 post-injury (164), whereas non-survivors of blunt trauma presented with significantly lower IL-33 concentrations 24-72 hours post-injury when compared to survivors (166). Acting as a decoy receptor and endogenous inhibitor of IL-33, elevated levels of soluble ST2 (sST2) have been detected in the circulation of trauma and burns patients in the days and weeks following injury (49, 165–167), with one prospective study measuring the highest concentrations of sST2 12-24 hours after the peak in IL-33 levels, a delay that was suggested would allow IL-33 to modulate systemic inflammatory responses (49). In respect to clinical outcomes, significantly higher concentrations of sST2 have been detected at several post-injury time points (hospital admission to day 5) in patients who develop pulmonary complications or succumb to their injuries (165, 166)

Suggesting a potential link between raised IL-33 levels and post-trauma immunosuppression, significantly higher concentrations of this DAMP have been measured in plasma samples acquired during the early (4-24 hours) and acute (day 4) post-injury phase from patients that develop HAIs when compared to those who do not (49). Mechanistically, this relationship could potentially be explained by IL-33 activating ILC2 cells and neutrophils to produce the type 2 cytokines IL-4, IL-5 and IL-13, whose plasma levels are significantly elevated in injured patients that experience HAIs (49, 168). Supporting this idea, positive associations have been reported between circulating concentrations of IL-33, IL-4, IL-5 and IL-13 in severely injured individuals (49), whilst in rodent models of trauma haemorrhage, IL-33 signalling is critical for both the post-injury expansion of ILC2 cells and the production of IL-5 by neutrophils (49). Recently, in the context of viral infections, IL-33-mediated production of IL-13 by ILC2 cells was shown to induce arginase-1 expression in neutrophils, the significance of which was demonstrated in neutrophil:T cell co-culture systems, where arginase-1 positive neutrophils significantly inhibited T cell proliferation (50). Relating these observations to traumatic injury, increased arginase-1 expression and activity has been reported in CD16^+^ granulocytes isolated from blunt/penetrative trauma patients for up to two weeks post-injury (169).

By inducing the expansion of IL-10 secreting M2 macrophages, and promoting the accumulation of regulatory T cells (Tregs), IL-33 has been proposed as a potential mediator of long-term immunosuppression in murine models of sepsis (48). Providing clinical relevance, significantly higher concentrations of IL-33 and IL-10, as well as increased frequencies of Tregs, have been detected in the circulation of sepsis survivors sampled 5-10 months post-diagnosis when compared to age and sex-matched controls (48). Thus, *could a similar mechanism underlie the development of PICS in critically-injured subjects?* With the potential to identify therapeutic targets for the treatment of this debilitating condition, future studies that prospectively measure the circulating levels of IL-33, its associated cytokines (e.g. IL-4, IL-5, IL-10 and IL-13) and the frequencies of Tregs in the months following major trauma are warranted. It is of interest that significantly elevated serum concentrations of IL-4, IL-5 and IL-13 have been reported in burns patients for up to three years post-injury (170).

#### 2.2.3 HMGB-1

HMGB-1 is a nuclear residing DNA-binding chaperone protein that regulates gene transcription. Entering the circulation as a consequence of necrosis, pyroptosis or active secretion through a secretory vesicular pathway (171–173), HMGB-1 concentrations are significantly elevated in the minutes, hours and days following major trauma (32, 174, 175). Positively associated with injury severity, base deficit and pro-inflammatory mediators (e.g. MPO, IL-6, IL-8), elevated levels of HMGB-1 have been linked to such poor clinical outcomes as MODS, organ injury and mortality (32, 174, 175). Recognised by several PRRs, which include TLR2, TLR4, TREM-1 and RAGE, HMGB-1 can both activate and inhibit immune cell function, with these opposing traits dependent upon its redox state. The immune suppressive actions of HMGB-1 arise from the terminal oxidation of three cysteine residues located within its DNA binding domains and acidic tail (159)

Like many other DAMPs, exposure to HMGB-1 blunts the pro-inflammatory responses of monocytes. Attributed to impaired activation of NFκB, resulting from reduced phosphorylation and degradation of IκBα, HMGB-1 preconditioning decreases TNF production by differentiated THP-1 cells and bone marrow-derived macrophages challenged with the TLR agonists LPS and lipoteachoic acid (51, 52). For the induction of endotoxin tolerance, HMGB-1 signalling through the RAGE receptor appears to be critical since pre-treatment with HMGB-1 failed to impair TNF production by LPS stimulated macrophages isolated from RAGE^-/-^ mice (51, 52). Contributing to further innate immune dysfunction, HMGB-1 reduces macrophage phagocytic activity (53), promotes the release of immature monocytes from the bone marrow (176) and inhibits neutrophil anti-microbial responses (54). Compared to vehicle controls, neutrophils treated with HMGB-1 exhibit decreased ROS production upon stimulation with PMA, an impairment that is associated with reduced phosphorylation of the NADPH oxidase subunit p40phox (54). In terms of clinical applicability, culturing neutrophils isolated from healthy controls with plasma from critically ill septic shock patients has been shown to significantly decrease their ROS production (54). Suggesting a role for HMGB-1 in mediating this effect, the addition of a neutralising anti HMGB-1 antibody to patient plasma restored neutrophil anti-microbial function (54). Whether similar mechanisms contribute to post-injury immunosuppression is unclear, but in a cohort of 88 blunt trauma patients plasma HMGB-1 levels were significantly higher within 4 hours of injury in those who later developed a HAI (168).

In a study that aimed to investigate the effect of HMGB-1 on T cell-mediated immunity following thermal injury, Huang et al. reported a profound effect of this DAMP on both CD4^+^ T cells and Tregs in rats (92). Compared to those isolated from sham controls, the authors found that post-burn, T cells from rodents subjected to a 30% TBSA burn exhibited reduced proliferative activity and skewed polarisation towards a Th2 phenotype, whilst Tregs produced greater amounts of IL-10 and expressed higher levels of RAGE (92). Implying a role for HMGB-1 in these responses, administration of an anti-RAGE antibody 6- and 24-hours post-burn injury reversed the burn-induced changes in both T cell function and phenotype (92). Supporting and expanding upon these results, a subsequent *in vitro* study found, in a TLR4 or RAGE dependent manner, that HMGB-1 treatment inhibited IFN-γ production by human CD4^+^ T cells and promoted the survival, migration and suppressive activity of Tregs (55).

Alongside direct modulation of immune cells, HMGB-1 may promote systemic immunosuppression post-injury by promoting the expansion of MDSCs. MDSCs suppress innate and adaptive immune responses via a number of mechanisms, which include the production of IL-10 and prostaglandin E_2_, the sequestration of cysteine, the breakdown of arginine and the generation of ROS (177, 178). Across several studies, HMGB-1 has been shown to promote the differentiation, survival, migration and activation of MDSCs (56). In the context of traumatic injury, elevated frequencies of MDSCs have been detected in the bone marrow, blood and spleen of mice subjected to peripheral tissue trauma, with this elevation prevented by post-injury administration of a blocking anti-HMGB-1 antibody (93). An expansion in spleen resident MDSCs has also been reported in a murine model of neurological trauma, which was accompanied by a significant increase in arginase-1 (ARG-1) expression in CD11b^+^ monocytes (176). A reduction in MDSC frequency in RAGE^-/-^ mice, and the absence of the trauma-induced increase in ARG-1 expression in monocytes isolated from mice pre-treated with an anti-HMGB-1 antibody, demonstrates a role for this DAMP in mediating systemic immunosuppression (176).

**Figure 1:**
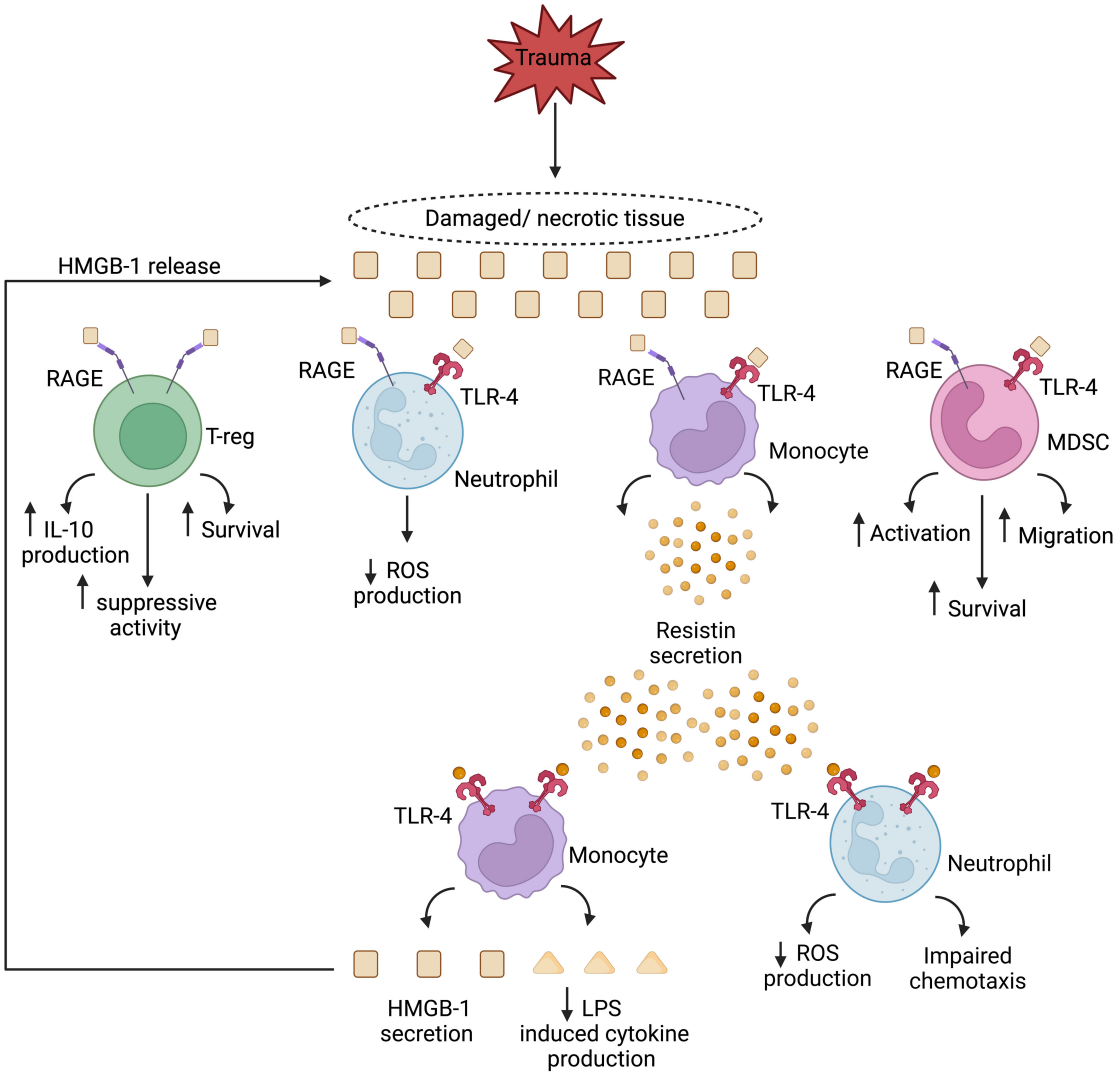
Theoretical model of a HMGB-1 and resistin-induced vicious cycle of immunosuppression in the setting of sterile injury. Passively released from damaged/necrotic tissue, the nuclear-derived damage associated molecular pattern high mobility group box-1 (HMGB-1) modulates the functions of innate and adaptive immune cells. Via binding to the pathogen recognition receptors toll like receptor-4 (TLR-4), and receptor for advanced glycation end products (RAGE), HMGB-1 directly inhibits neutrophil anti-microbial functions and enhances the suppressive activities of regulatory T cells and myeloid derived suppressor cells (MDSCs). Monocytes activated by HMGB-1 secrete resistin, an inducer of endotoxin tolerance, and a negative regulator of neutrophil chemotaxis and reactive oxygen species (ROS) production. In a self-perpetuating feedback loop, resistin, through TLR-4 signalling, promotes the secretion of HMGB-1 by monocytes, culminating in a localised immune suppressive environment at the site of tissue injury. IL-10, Interleukin-10; LPS, Lipopolysaccharide. Figure was generated at Biorender.com.

Human monocytes treated with HMGB-1 secrete the peptide hormone resistin (179). Shown *in vitro* to inhibit both neutrophil chemotaxis and ROS generation (180–182), and to suppress TNF production by E.coli or LPS challenged monocytes (183, 184), elevated resistin concentrations have been measured in the circulation of TBI and thermally-injured patients (185–188). Furthermore, and with the potential to create a perpetual cycle of immunosuppression, monocytes treated with physiological concentrations of resistin secrete HMGB-1 and upregulate their expression of TLR4 (189). Thus, systemically, and at sites of tissue damage, HMGB-1 and resistin may combine to create an immune suppressive environment that predisposes the hospitalised trauma patient to secondary infections **(** **Figure 1** **)**. Crucially, this cycle may be amenable to therapeutic intervention, with studies showing that *in vitro* hemoadsorption and nucleic acid scavenging microfiber meshes can deplete resistin from serum samples of critically-ill patients (190) and HMGB-1 from supernatants of necrotic cells (191). In the case of resistin, the correction of hyperresistinemia restored neutrophil migration and bacterial killing capacity in serum co-culture assays (190).

### 2.3 MtDAMPs

#### 2.3.1 ATP

ATP is released non-specifically from necrotic cells, or actively by phagocytes, endothelial cells and apoptotic cells in response to inflammatory challenge, hypoxia or oxidative stress, with its secretion mediated by vesicular exocytosis or through pore-forming channels such as connexins and pannexins (192). Murine models of sepsis have reported elevated plasma concentrations of ATP within two hours of cecal ligation and puncture (193), whilst resuscitation protocols may exacerbate direct trauma-induced increases in circulating ATP levels since *in vitro* exposure to hypertonic saline induces ATP secretion from neutrophils and T cells (194, 195).

Although not reported by all studies (196, 197), the current literature points towards ATP as a DAMP with potent pro-inflammatory properties and an initiator of the SIRS response. For example, signalling through the P2 family of purinergic receptors, ATP (1): promotes inflammasome-mediated secretion of IL-1β by LPS primed macrophages (198) (2); triggers TNF, IL-6 and IL-1α production by macrophages (199); (3) induces T cell activation and IL-2 production (200); (4) augments fMLP-induced superoxide production by neutrophils (194); (5) stimulates neutrophil degranulation, aggregation and adherence to endothelium (201). Moreover, in a murine model of LPS-induced shock, systemic administration of the ATP hydrolase apyrase reduced the cytokine storm, immune cell infiltration into peripheral organs and apoptotic cell death that was observed in vehicle-treated littermates (202). Removal of ATP has therefore been proposed as a potential strategy for dampening the SIRS response and its associated toxicity in the setting of sterile inflammation (202).

In the extracellular environment, ATP levels are regulated by the ectonucleotidases CD39 and CD73. Expressed by various cell types that include endothelial cells, monocytes, lymphocytes, neutrophils and Tregs, CD39 converts ATP and ADP into AMP, which is subsequently degraded into adenosine by CD73 (203, 204). A potent anti-inflammatory molecule, adenosine inhibits neutrophil phagocytosis, ROS production, degranulation and cytokine production (57), suppresses T cell migration, adhesion and IFN-γ production (58), downregulates antigen presentation and T cell priming by DCs (59), reduces TLR-induced pro-inflammatory cytokine production by macrophages (205) and decreases NK cell cytotoxicity (58). Mechanistically, this suppression is mediated through the A2 receptor family, and in particular the A_2A_ subclass, whose signalling pathways result in the production of cyclic AMP and activation of protein kinase A (57, 59). Interestingly, a recent study reported a trend towards an increased frequency of CD8^+^ CD39^+^ T cells in the circulation of trauma patients at days 0/1 post-injury when compared to control patients undergoing elective surgery (206). Immune phenotyping revealed these T cells exhibited signs of immune exhaustion, with increased expression of the markers PD-1 and TIM-3 (206). These changes were accompanied by a trend towards reduced granzyme B content, suggesting potentially reduced cytotoxic capacity (206). Prominent in trauma patients with SIRS, it was speculated that an abundance of immunologically exhausted CD8^+^ CD39^+^ PD-1^+^ Tim-3^+^ T cells may contribute to the state of systemic immune dysfunction that increases the susceptibility of trauma patients to infection (206).

Data are emerging that implies potential interplay between ATP, mtDNA, HSPs and heme metabolism in mediating post-trauma immunosuppression. Suggesting that recognition of mtDNA may enhance T cell mediated conversion of ATP/ADP to its immune suppressive metabolite adenosine, a small cohort study of 11 trauma patients reported a positive association between plasma mtDNA levels and the frequency of CD8^+^CD39^+^ T cells heckler (206). In a series of *in vitro* experiments, Haschemi et al. demonstrated a positive feedback loop between adenosine and HSP32 in suppressing LPS-induced TNF production by macrophages (60). Through activation of the A_2A_ receptor, the authors found adenosine induced expression of heme oxygenase (HSP32) in macrophages (60). Degradation of heme by HSP32 resulted in the production of carbon monoxide, which increased the sensitivity of macrophages to the anti-inflammatory effects of adenosine by increasing surface expression of the A_2A_ receptor (60). Relating these findings to trauma, monocytes isolated from injured patients with severe SIRS have been shown to exhibit increased expression of HSP32 and impaired TNF production following bacterial stimulation (115).

#### 2.3.2 MtDNA

In a seminal study published in 2010, Zhang and colleagues provided mechanistic insights into how sterile traumatic injury results in systemic inflammation and organ dysfunction (33). Passively released from necrotic/damaged tissues, and actively secreted by immune cells (35, 207, 208), they showed that mtDNA, whose levels are acutely and persistently elevated post-trauma (31, 33, 76, 77, 209) is a potent activator of neutrophils, with exposure to this DAMP triggering IL-8 production and activation of MAPKs (33). Subsequent studies have confirmed these observations and demonstrated that mtDNA also induces NET formation and neutrophil degranulation in a TLR9 dependent manner (72, 210, 211). Linking the immunostimulatory properties of mtDNA to systemic inflammation and organ dysfunction, injection of mtDNA into rodents results in hepatic inflammation (210), whilst in models of traumatic injury and shock, plasma mtDNA levels positively associate with lung MPO levels and circulating urea and IL-6 concentrations (76). Providing clinical relevance to these findings, prospective studies of critically-injured subjects have shown plasma mtDNA levels in the early and acute post-injury phase is an independent predictor of SIRS (77, 212). Further, mtDNA load was significantly higher in non-survivors versus survivors as well as patients who develop such secondary complications as ARDS and MODS (76, 78, 213). Interestingly, circulating cell-free mtDNA (cfmtDNA) exists in two forms, a low molecular weight form and a larger form, with the latter comprising 95% of cfmtDNA (214, 215). Comparing these two fractions in plasma samples from 25 major trauma patients, Briggs et al. demonstrated that it was the levels of low molecular weight cfmtDNA that were positively associated with poor clinical outcomes such as ICU length of stay, ventilator use and duration of MOF (216). When interpreting their findings, the authors suggested that the larger forms of cfmtDNA are membrane encapsulated and therefore unable to bind to TLR9 and trigger immune responses (216).

Alongside its pro-inflammatory properties, data are emerging that demonstrates mtDNA is a potent immune suppressor. Treatment of human monocytes with mtDNA inhibits, in a TLR9 dependent manner, their production of IL-1β, IL-6 and TNF upon subsequent stimulation with LPS (61, 62). This mtDNA-induced reduction in cytokine production was attributed to impaired activation of NF-κB signalling and the induction of IRAK-M, a negative regulator of TLR signalling (61). In the setting of sterile neurological trauma, circulating levels of mtDNA are significantly elevated in patients who develop HAIs, with *ex vivo* studies showing that pre-treatment of monocytes isolated from HCs with sera from these patients reduced LPS-induced TNF production, an impairment that was reversed when monocytes were treated with the TLR9 antagonist ODN prior to serum incubation (62). In addition to monocytes, mtDNA induces functional tolerance in neutrophils, with prior exposure to this DAMP inhibiting neutrophil chemotaxis, phagocytosis and bacterial killing (35, 63). A critical step in each of these defence mechanisms is reorganisation of actin filaments, a process that involves activation of such cytoskeletal proteins as the F-actin binding protein cortactin (CTTN). Through activation of G protein coupled receptor kinase 2 (GRK2), mtDNA treatment suppresses CTTN activation by promoting its deacetylation by histone deacetylases (HDACs) (63). Coinciding with elevated plasma mtDNA levels, activated GRK2 and reduced CTTN acetylation have been detected in neutrophils isolated from trauma patients (63). Demonstrating the potential relevance of these observations to clinical outcomes, GRK2 activity and CTTN acetylation, at days 1-6 post-injury, were increased and decreased respectively in neutrophils of trauma patients who later developed HAIs (63).

With potential implications for the efficiency of adaptive immune responses, mtDNA negatively impacts upon DC function. Actively engulfed from the extracellular environment, elevated cytoplasmic mtDNA levels have been measured in DCs isolated from septic mice and in bone marrow-derived DCs challenged with LPS and mtDNA *in vitro* (64). Through activation of the cGAS-stimulator of interferon genes (STING) pathway, JAK-1/2 and STAT3 signalling, the accumulation of cytoplasmic mtDNA inhibited LPS-induced expression of the co-stimulatory molecules CD40, CD80 and CD86 (64). Accompanied by reduced production of IL-12p70 and enhanced synthesis of IL-10, these phenotypic changes were associated with an impaired ability of DCs to promote the proliferation of CD4^+^ T cells in co-culture systems (64). Bearing unmethylated CpG islands, mtDNA is a ligand for the surface expressed and endosomal residing PRR TLR9. In murine models of thermal injury, CD4^+^ T cells cultured with TLR9 activated DCs isolated from burn-injured mice were found to secrete markedly lower levels of Th1 and Th17 cytokines when compared to T cells cultured with DCs from sham controls (217). This shift in T cell polarisation was associated with increased production of IL-10 and reduced secretion of IL-6, IL-12p70 and TNF by TLR9 challenged DCs post-burn (217).

As a DAMP that possesses both immune stimulatory and inhibitory properties, mtDNA and/or its associated signalling pathways have been discussed as possible therapeutic targets for the treatment of post-traumatic SIRS, ARDS and immunosuppression (35, 63, 76, 78, 213). Focussing on the latter, preclinical studies have demonstrated the potential immune enhancing effects of GRK2 and HDAC inhibitors (63). Administered 30 minutes after a liver crush injury and tracheal inoculation of *S.aureus*, a combined therapy of valproate, a HDAC inhibitor, and the GRK2 inhibitor paroxetine restored levels of bacterial clearance to those recorded in uninjured controls and increased survival rates (63). Importantly, for its potential translation into the hospital setting, the treatment regimen was not associated with any evidence of organ injury or failure (63).

#### 2.3.3 Mitochondrial-derived N-formylated peptides

Mitochondria synthesise 13 formylated peptides that share molecular similarities with the formyl peptides derived from bacteria. All but one of these formylated peptides, namely Cox1, have been detected in the circulation of trauma patients, with concentrations of ND6, a subunit of mitochondrial NADH dehydrogenase, significantly elevated within 1-hour of injury and remaining so for up to 72 hours (30, 66).

Detected by surface expressed formyl peptide receptor (FPR) 1 and FPR2 (67), mtFPs are neutrophil chemoattractants, with the proteins that most closely resemble bacterial-derived formylated peptides, namely ND3, ND4, ND5 and ND6 possessing the greatest chemotactic potency (66). Data generated by several studies have demonstrated that neutrophils pre-treated with ND6 exhibit reduced calcium fluxes, chemotactic responses and ROS production upon secondary stimulation with other GPCR agonists such as IL-8, leukotriene B4 (LTB_4_) and fMLP (30, 65–68). Contributing to this heterologous receptor desensitisation and suppression in anti-microbial functions is an ND6-mediated downregulation of GPCRs, for which activation of GRK2 is a potential underlying mechanistic explanation (63, 65, 66, 218).

Associated with reduced bacterial clearance, a peripheral fracture injury in animal models has been shown to reduce neutrophil recruitment to the lung following a pulmonary contusion (219). As femoral fracture reamings are a rich source of mtDAMPs (91), it has been proposed that the increased susceptibility of trauma patients to HAIs may arise in part from mtDAMPs impairing host resistance by attracting neutrophils towards distal injury sites and away from regions of pathogenic challenge (219, 220). Supporting this hypothesis, intra-peritoneal injection of mitochondrial debris was shown to markedly attenuate neutrophil migration to the lung following a pulmonary contusion (220). A role for mtFPs in mediating this effect is suggested by the results of another animal study in which administration of the FPR1 receptor antagonist cyclosporin H, alongside mtDAMPs, significantly improved bacterial clearance in the lung following intra-tracheal injection of *S.aureus* (218).

Implicated in the capture, neutralisation and elimination of pathogens, the generation of NETs is significantly reduced post-injury (30, 219). In a series of *in vitro* and *ex vivo* experiments, we attributed this impairment to a mtFP-induced dysfunction in neutrophil metabolism (30). Compared to those isolated from healthy controls, neutrophils obtained from trauma patients, as early as 1-hour post-injury, exhibited reduced glucose uptake and breakdown, two prerequisites for NET formation (30). Replicating these impairments *in vitro*, we showed that treatment of control neutrophils with whole mtDAMP preparations, but not purified mtDNA, inhibited PMA-induced NET production, a defect that was associated with reduced aerobic glycolysis and a mtFP dependent activation of AMP-activated protein kinase, a negative regulator of NET formation (30).

As a DAMP that suppresses a range of neutrophil anti-microbial functions, studies have investigated whether mtFPs may represent a potential therapeutic target to combat post-trauma immunosuppression. Several studies have shown that pre-treatment of neutrophils with FPR1 inhibitors not only prevents mtFP-induced impairments in calcium mobilisation and chemotactic responses (65, 66, 68) but also reduces ND6-mediated downregulation of GPCRs (65, 218). Another strategy under consideration is the intra-tracheal instillation of neutrophils, which aims to overcome the ability of mtFPs generated at regions of tissue injury to direct neutrophils away from sites of pathogenic challenge. In preclinical models of pseudo fracture and bacterial inoculation, intra-tracheal instillation of exogenous bone marrow derived murine neutrophils or neutrophils isolated from human blood samples has been shown to prevent the establishment of and/or treat pneumonia by reversing the mtDAMP-induced impairment in bacterial clearance from the lungs (68, 221, 222). Encouragingly, no adverse effects in respect of organ damage have been detected post-instillation (221, 222). Furthermore, and relevant to its potential translation to the clinic, preliminary data suggest that cryopreserved neutrophils are just as effective as freshly isolated neutrophils in promoting bacterial clearance from the lung in the presence of mtDAMPs (222).

## 3 Conclusions

The detection of DAMPs in blood samples obtained from critically-injured patients in the pre-hospital setting offers a mechanistic explanation for the simultaneous induction of the SIRS and CARS response that occurs within minutes of injury (15, 31). Indeed, alongside their renowned pro-inflammatory properties, cytosolic, nuclear and mitochondrial-derived DAMPs are now recognised as potent immune suppressors, impairing the anti-microbial functions of innate and adaptive immune cells either directly or by driving the expansion and differentiation of Tregs and MDSCs. Exacerbating both the local and systemic immune suppressive environments created by the instantaneous release of DAMPs from injured tissues, are secondary insults such as fluid resuscitation, surgical interventions and transfusions with aged blood cell products, which either induce the release of endogenous DAMPs or are themselves a source of exogenous DAMPs. Furthermore, alongside pre-existing DAMPs, a novel class of suppressive inducible DAMPs, termed SAMPs, have recently been described and suggested to activate hyper-resolution responses within minutes of the SIRS response being initiated (82).

**Figure 2:**
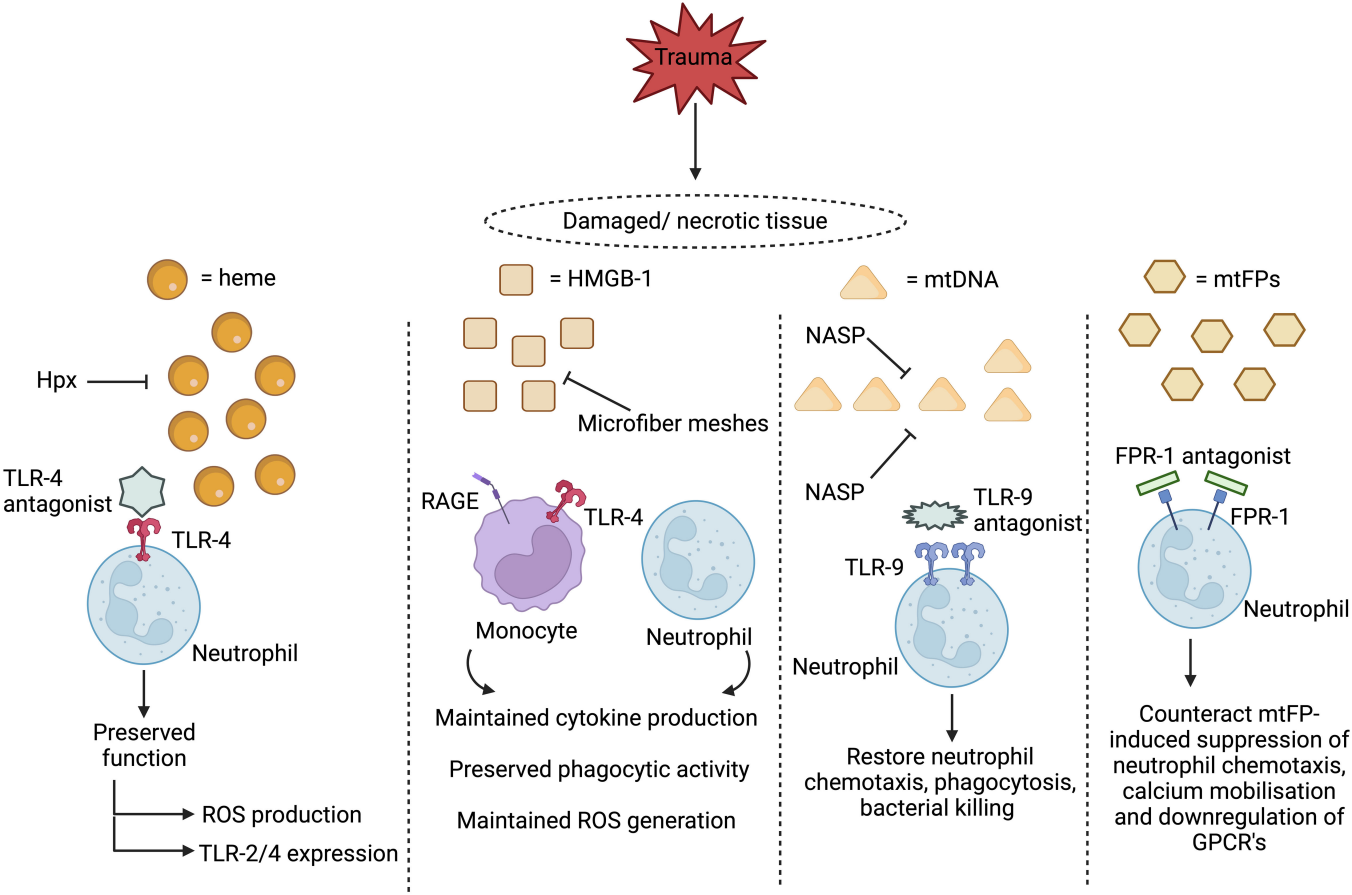
Proposed therapeutic strategies for treating damage associated molecular pattern (DAMP)-induced immunosuppression. Two therapeutic approaches under consideration for preventing and/or reversing DAMP-induced immunosuppression are to scavenge circulating DAMPs or to inhibit their activation of immune cells. Restoring circulating levels of hemopexin (Hpx), a heme scavenging protein whose plasma concentrations are reduced post-injury, may help combat heme-induced suppression of neutrophil anti-microbial functions, whilst the use of microfiber meshes or nucleic acid scavenging polymers (NASPs) to reduce the systemic load of circulating mtDNA and HMGB-1 could alleviate the post-trauma impairments reported in monocyte and neutrophil function. Showing promise in *in vitro* studies, blocking DAMP-induced activation of neutrophils through the use of toll-like receptor-9 (TLR-9) or formyl peptide receptor-1 (FPR-1) antagonists prevents the induction of functional tolerance to subsequent secondary stimulation with inflammatory agonists or microbial-derived proteins. HMGB-1, High mobility group box-1, mtDNA, Mitochondrial-derived DNA; mtFPs, Mitochondrial-derived N-formylated peptides; RAGE, Receptor for advanced glycation end products; ROS, Reactive oxygen species; TLR, Toll-like receptor. Figure was were generated at Biorender.com.

Currently, very few prospective studies have investigated whether post-trauma elevations in circulating DAMPs are associated with and/or predictive of the development of HAIs. However, studies in other cohorts of critically-ill patients suggest such a relationship may exist (65, 223). If translatable to trauma patients, then the question arises of whether DAMPs and/or their associated signalling pathways represent feasible therapeutic targets for the prevention and/or treatment of secondary infections post-injury. Scavenging circulating DAMPs via the use of microfiber meshes or polymers, or inhibiting ligand-receptor interactions through delivery of receptor antagonists, are strategies under consideration for treating the secondary complications associated with the post-traumatic SIRS response that may also be suitable for combating the immune suppressive actions of DAMPs **(** **Figure 2** **)** (76, 191). However, the timing of such treatments would need to be carefully considered as it must not be forgotten that the purpose of DAMP release is to alert the body to tissue damage. Thus, a fine balance will exist for achieving an optimal immune response, with excessive or inappropriate removal of DAMPs, or inhibition of immune cell signalling, having the potential to negatively impact upon the DAMP-induced initiation of inflammatory responses that are critical for the regenerative processes of tissue repair and healing.

## Author contributions

EH and JH conceived and wrote the manuscript. JL critically appraised and revised the manuscript. All authors contributed to the article and approved the submitted version.
